# Supplementary material for: Characterisation of biomarkers of intestinal barrier function in response to a high fat/high carbohydrate meal and corticotropin releasing hormone
Source: PLoS One. 2024 Feb 26;19(2):e0294918. doi: 10.1371/journal.pone.0294918 (PMC10896497; doi:10.1371/journal.pone.0294918)
Supplement: S1 Fig — (DOC) [file pone.0294918.s002.doc]

**
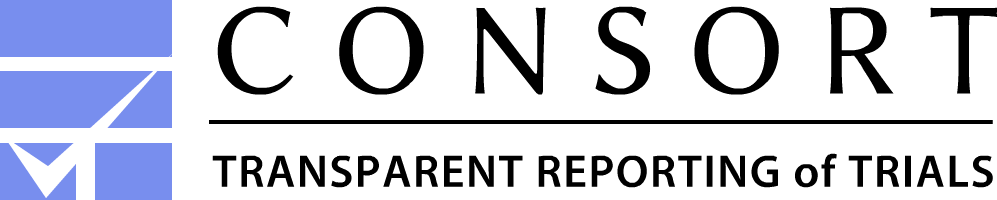
**

**CONSORT 2010 Flow Diagram**

**Allocation**

**Analysis**

**Follow-Up**

**Enrollment**

Assessed for eligibility (n= 15 )

Excluded (n= 5 )

♦  Not meeting inclusion criteria (n= 2 )

♦  Declined to participate (n= 0 )

♦  Other reasons (n= 3 )

Analysed (n= 10 )
♦ Excluded from analysis (give reasons) (n= 0 )

Lost to follow-up (give reasons) (n= 0 )

Discontinued intervention (give reasons) (n= 0 )

Allocated to intervention (n= 10 )

♦ Received allocated intervention (n= 10 )

♦ Did not receive allocated intervention (give reasons) (n= 10 )

Lost to follow-up (give reasons) (n= 0 )

Discontinued intervention (give reasons) (n= 0 )

Allocated to intervention (n= 10 )

♦ Received allocated intervention (n= 10 )

♦ Did not receive allocated intervention (give reasons) (n= 10 )

Analysed (n= 10 )
♦ Excluded from analysis (give reasons) (n= 0 )

Randomized (n= 10 )
