# Supplementary material for: Characterisation of biomarkers of intestinal barrier function in response to a high fat/high carbohydrate meal and corticotropin releasing hormone
Source: PLoS One. 2024 Feb 26;19(2):e0294918. doi: 10.1371/journal.pone.0294918 (PMC10896497; doi:10.1371/journal.pone.0294918)
Supplement: S1 File — (DOCX) [file pone.0294918.s003.docx]

**Intestinal Barrier Function Changes in Response to Administration of Corticotropin Releasing Hormone and High Fat Diet**

**Background**

| The last decade has seen a number of studies supporting the theory of chronic intestinal barrier perturbation in the pathogenesis of IBD and many other human diseases.^1–4^ Stress – a state which evokes a ‘fight or flight’ or sympathetic response from the human organism - has been implicated in the propagation of inflammation in patients with inflammatory bowel disease (IBD). Indeed, the risk of relapse in both Crohns disease (CD) and Ulcerative colitis (UC) increases in the presence of both acute and chronic stress. ^5–7^ The mechanism proposed for this phenomenon includes the ability of stress to induce a breakdown in intestinal barrier function via mast cell- and corticotropin releasing hormone (CRH)-dependent pathways. ^8^ This intestinal barrier perturbation has been demonstrated experimentally in humans using stress paradigms which could be equated to the relatively mild stressful events experienced in every-day life, such as public speaking.^9–11^, although replicating this has practical difficulties in a research setting. Another established way to induce intestinal stress is through diet.  The autonomic nervous system (ANS) appears to be playing a crucial role in stress-induced changes in intestinal permeability. Multiple in vitro and in vivo murine model studies have confirmed that both acute and chronic stress (induced in a mouse by various stress paradigms such as restraint and water emersion) can promote increased intestinal permeability. The underlying mechanism seems to be via the disruption of tight junction proteins along the paracellular space of the intestinal epithelium.^15,16^ The disruption of tight junctions in these stress paradigm models are dependent on acetylcholine and CRH. ^11,17^ This effect on intestinal permeability appears to recover within 4 days in murine models.^18^ Similar changes have been described in human models of acute stress. Changes in intestinal permeability induced by acute stress can be mimicked by peripheral administration of CRH.^19^  Corticotropin releasing hormone (CRH) is naturally secreted from the healthy human hypothalamus during periods of stress. Intravenously administered CRH possesses the advantage of having a short half-life and is routinely clinically utilised in the diagnosis of Cushing’s syndrome.^12,13^ Recent evidence has shown that injecting CRH intravenously reliably and significantly increases intestinal permeability in healthy volunteers with no significant adverse effects.(12) The timeline of this increase, and its effect on the serum intestinal permeability measurements have not been established.  Food-induced stress has also shown to temporarily impair barrier function in healthy subjects with a standardised ‘high fat high carbohydrate’ meal. This challenge meal, comprising 50-60 g fat and 80 g carbohydrate, caused bacterial translocation indicated by increased sCD14 and LBP^14^, although it is not known if these changes are maintained beyond four hours. This standardised meal has been used in research setting in many studies and is accepted as gold standard. However, using food as a model to represent IBD has its limitations. Food is complex in its composition and has many confounding nutrient factors, background diet is rarely considered and may impact outcomes, blinding is difficult, recruitment of study participants will automatically exclude those with dietary restrictions or certain food dislikes and using food-induced stress is not appropriate for trials investigating dietary therapies. Furthermore, it is difficult to translate the findings into real world, which involves constant ingestion of food. We need a more reliable model of experimentally inducing intestinal barrier dysfunction in humans.  Several serological and urinary markers have been validated as useful tools in measuring intestinal  permeability changes in vivo. These include lipopolysaccharide-binding protein (LBP), soluble CD-14  (sCD14), syndecan-1 and intestinal-type fatty acid-binding protein (I-FABP) in the serum. Because  sCD-14 and LBP are part of the LPS inflammatory signalling pathway, their levels in serum are believed  to be objective markers for systemic immune activation and damage to gut epithelium.^14,20^.  I-FABP is an intracellular protein expressed in the epithelial cells of the mucosal layer of the small  and large intestine tissue and is prone to leakage into the blood stream from the enterocytes when  intestinal mucosal damage occurs.^21^ Syndecan-1 is a key component of the glycocalyx layer of the  intestinal endothelial surface.^21^ Syndecan-1 is crucial to the maintenance of the intestinal epithelial  barrier function^22^ and its release into the circulation indicates perturbation of the intestinal barrier.^23,24^  While I-FABP and syndecan-1 are preformed and would be rapidly released on intestinal injury in association with acute stress, such as a single meal (as above) or CRH administration, the validity of the serum/plasma markers, LPB and sCD14, requires critical examination. The response of these markers is dependent upon new protein synthesis in response to cytokine release (specifically IL-1, IL-6 and TNFα) related to increased LPS/endotoxin exposure following changes in barrier function. Since such production is transcriptionally regulated, there will be a delay in detecting a rise in the concentrations of LPB and sCD14. In rabbits stimulated with silver nitrate, transcription had a delay of up to 2 hours. Subsequent LBP levels in serum were elevated at 4 hours after the stimulus and were maximal at 24 hours.^25^ The studies examining LPB and sCD14 after a high fat meal had changes observed within the first 2 hours.^14^ This is not easy to reconcile with new induction of protein synthesis and might reflect washing out of hose proteins from the intestinal mucosa via increased lymphatic flow, as previously shown for hyaluronic acid.^26^ Whatever the case, the question of the time course of events requires clarification.  We aim to develop a model of intestinal permeability perturbation in healthy human volunteers with both CRH and a standardised high fat high carbohydrate meal. This will allow for future studies to experimentally test therapeutic efficacy of CRH on the intestinal barrier function.  **AIMS:**   1. To develop models of intestinal permeability perturbation in healthy human volunteers utilising 2 strategies  - Physiological stress induction by intravenous CRH injection - Clarification and further exploration of the stress effect of a standardised high-fat high-carbohydrate (HFHC) meal on intestinal barrier dysfunction. This data will be used in future studies to dissect out the particular dietary components within this meal which specifically induce permeability changes.  1. To characterise the models in terms of optimal timing of blood sampling circulating markers of intestinal barrier dysfunction   **HYPOTHESES:**   1. Both CRH and the high-fat high-carbohydrate meal will increase intestinal permeability over a normal saline injection as measured by serum markers of intestinal permeability (I-FABP, Syndecan-1, CD14 and LBP). 2. There are differences in optimal timing of the measurement of circulating markers.     **Project Design**  This will be a single centre, randomised, placebo-controlled cross-over study to investigate the effect of CRH and a HFHC meal on intestinal permeability. (See ***Figure 1***).  ***Figure 1***  ***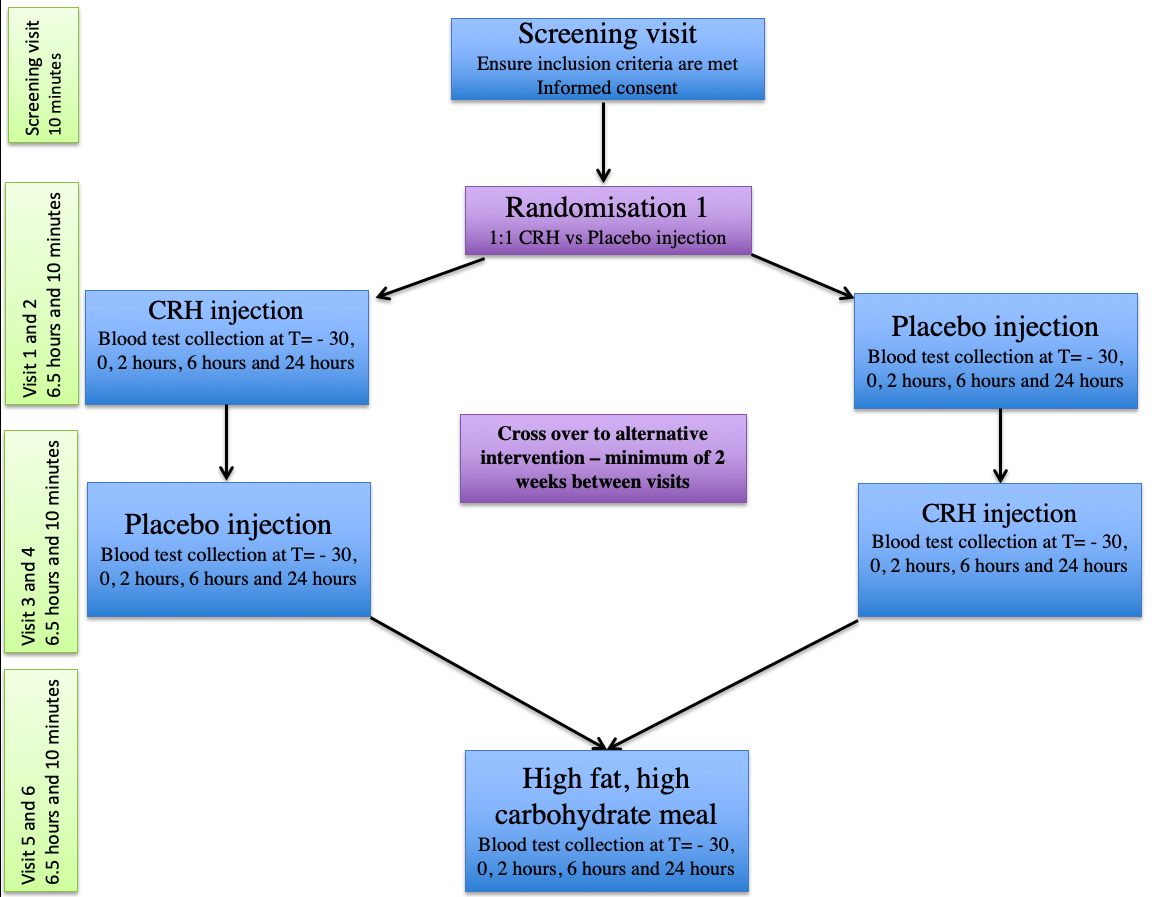***  **Inclusion criteria:**   - Healthy males and females between the ages of 18 – 65 - Participants who are able to give informed consent - Volunteers should be able to attend Monash University, Alfred Campus on 7 occasions   **Exclusion Criteria**   - Inclusion criteria not met - Past medical history of diabetes (type 1 or 2) - Past medical history of inflammatory bowel disease, coeliac disease, a diagnosis of irritable bowel syndrome or significant gastrointestinal symptoms not otherwise medically diagnosed - Patients actively taking non-steroidal anti-inflammatory medications (NSAIDs) or corticosteroids - Probiotic or antibiotic intake over the past 2 months - Pregnant or breast feeding participants - Allergy or intolerance to the components of the proposed meals   **Recruitment process**  Participants will be recruited using advertising posters and word of mouth from the Monash University community.  **Study Procedures**  These are explained in ***Figure 2*** and in further detail below  ***Figure 2***  **Visit 1 and 2**   \| **Time (minutes)** \| **T = -30** \| **T = 0** \| **T = 2 hours** \| **T = 6 hours** \| **T = 24 hours** \| \| --- \| --- \| --- \| --- \| --- \| --- \| \| **Intervention** \| - Cannula insertion and initial blood sample \| - CRH or placebo injection - Further blood sample \| - Blood sample \| - Blood sample - Cannula removal \| - Blood sample   **END OF VISIT 1 AND 2** \|   **Visit 3 and 4**   \| **Time (minutes)** \| **T = -30** \| **T = 0** \| **T = 2 hours** \| **T = 6 hours** \| **T = 24 hours** \| \| --- \| --- \| --- \| --- \| --- \| --- \| \| **Intervention** \| - Cannula insertion and initial blood sample \| - High fat , high carbohydrate meal to participant - Further blood sample \| - Blood sample \| - Blood sample - Cannula removal \| - Blood sample   **END OF VISIT 3 AND 4** \|   *Participants will be asked to obtain from alcohol and intense exercise for 3 days prior to each of the study visits.*  **Screening visit**  Participants will be invited to meet with the study staff in order to ensure eligibility and seek informed consent. They will be informed whether or not they meet inclusion criteria at the end of the screening visit. They will be given a list of foods which are low in fat and carbohydrates. These are the only foods that they will be able to consume for the duration of the visits.  *All blood tests will involve 2x 10ml blood test tubes drown from the participants’ vein unless otherwise stated*  **Visit 1**   - Participants will, if they wish, have a light breakfast consisting of foods from a prespecified list which have a low fat and low carbohydrate content. - On presentation to the department, participants will be randomised to receive either a CRH or placebo injection. - They will then have a cannula inserted and have blood drawn. This will be the Time (T) = -30 minute blood test. - Participants will have a further blood test at the T=0 mark and then have either CRH or placebo injected into their cannula. - They will have further blood tests at the T=2 and T= 6 hour marks. - Participants will be asked to remain near the department for the duration of the study visit and will be offered a light hearted video to watch for the period of their visit. - After T=6 hours, the cannula will be removed and participants will be free to leave the department. - A low fat, low carbohydrate late lunch meal will be provided to the participants on completion of   this visit and instructions for how to prepare a low fat, low carbohydrate dinner will be given to them to follow for their evening meal.  **Visit 2**   - Participants will be asked to return the day after Visit 1 – at T = 24 hours post the initial CRH or placebo injection in order to have their final blood tests.   ***Visits 1 and 2 will be replicated during visits 3 and 4 with the alternative intervention.***  **Visit 5**   - Participants will be asked to present to Monash University, The Alfred Hospital campus. - They will have a cannula inserted and have blood taken. This will be the T = -30 sample - They will then receive the high fat meal. - ***Participants will not be informed of the nature of the meal that they are receiving*** - They will eat the meal and have the second blood test immediately after they have finished eating. This will be the T = 0 blood test. - They will have further blood tests at T = 2 and T = 6 hour mark, after which time they will have their cannula removed and they will be free to leave the department. - A low fat, low carbohydrate late lunch meal will again be provided to the participants on completion of this visit and instructions for how to prepare a low fat, low carbohydrate dinner will be given to them to follow for their evening meal.   **Visit 6**   - The participant will be asked to return at the 24 hour mark where they will have a further blood test performed.   **Outcome measures**  ***Primary outcome measures:***  Change in intestinal permeability over the different time points in response to CRH, placebo and meal challenge as measure by:   - - Serum I-FABP, Serum CD14, serum syndecan-1 and Serum LBP as measured by the ELISA   **Data Collection/Gathering**  After obtaining informed consent, data will be collected on the participants’ demographics. This will be stored in a secure, password protected database in a re-identifiable fashion. Paper records will be kept on site at Monash university in a secure location.  **Participant Safety and Withdrawal**  ***Risk management and safety***  ***The potential for intravenous CRH administration to cause side effects***  CRH is a hormone naturally produced in the human hypothalamus in response to stress. Its intravenous preparation is used routinely in clinical practice for the diagnosis of Cushings syndrome at a dose of 200 μg.^13^ CRH has rarely been associated with side effects such as flushing and tachycardia when administered at doses ***greater*** than 200 μg and its pharmacology has been thoroughly characterised in multiple previous studies.^13,16^ We are proposing to administer 100 μg, which is half of that used in routine clinical practice. Multiple studies have been performed on healthy human volunteers and higher at this dose of CRH with no reported side effects aside from *some minor facial flushing* in one study 30 minutes post administration.^13,15,19^ Participants will be monitored in the presence of medical staff throughout this period.  ***The standardised challenge meal***  The standardised meal will provide 56-60 g total fat, 82 g carbohydrate and 28-30 g protein and will consist of two sausage, egg and cheese sandwiches with two hash browns. This challenge meal has been used in similar studies.  ***Pain/discomfort/distress during interventions, particularly cannulation for CRH administration and blood taking***  Participants will all undergo informed consent where a member of the investigator team will be able to guide them through the interventions. They will be reassured and informed that they can stop being involved in the study at any point of their choosing. Cannulation, blood taking and CRH administration will be performed by an experienced medical practitioner. The cannula will remain in-situ for no longer than the duration of the visit (6.5 hours). This is well below the recommended 72 hours (or 3 days) of cannulation duration in Australian hospitals.  ***Withdrawals***  Participants are able to withdraw from the study at any point in time. The data will be collected up until the withdrawal from the study and included in the final analysis.  ***Replacements***  If a participant chooses to withdraw from the study prior to completion a further patient will be recruited until a total of 10 patients have completed the study.  **Storage of blood and data security**  ***Blood and Tissue Storage***  Two vials of serum will be stored in aliquots for further analysis. These samples will be assigned to the participant’s research code and will remain within the Monash University at the Alfred Hospital. After analysis has been completed the blood and tissue will be destroyed.  ***Data Security***  Electronic records of participant data will be kept securely with the principal investigator whilst paper records will be kept at Monash university. At the completion of the study all records will be kept with the principal investigator for at least 7 years. All electronic records will require password access and paper records will be in security tag accessible areas within locked storage units.  **Data Analysis**  Given that this is the first study investigating the timeline of intestinal permeability change in response to the described stimuli, a sample size of 10 participants has been chosen to initially explore these effects. The median values of the quantitative results of the ELISA will be compared between the 3 different conditions. Non parametric paired analyses will be performed and a statistical criterion of p<0.05 as significant will be adopted. | |
| --- | --- | --- | --- | --- | --- | --- | --- | --- | --- | --- | --- | --- | --- | --- | --- | --- | --- | --- | --- | --- | --- | --- | --- | --- | --- |
|  |  |

**References**

1. Söderholm JD, Peterson KH, Olaison G, et al. Epithelial permeability to proteins in the noninflamed ileum of Crohn’s disease? *Gastroenterology*. 1999;117(1):65-72. http://www.ncbi.nlm.nih.gov/pubmed/10381911. Accessed August 21, 2018.

2. Söderholm JD, Olaison G, Peterson KH, et al. Augmented increase in tight junction permeability by luminal stimuli in the non-inflamed ileum of Crohn’s disease. *Gut*. 2002;50(3):307-313. http://www.ncbi.nlm.nih.gov/pubmed/11839706. Accessed August 21, 2018.

3. Zeissig S, Burgel N, Gunzel D, et al. Changes in expression and distribution of claudin 2, 5 and 8 lead to discontinuous tight junctions and barrier dysfunction in active Crohn’s disease. *Gut*. 2007;56(1):61-72. doi:10.1136/gut.2006.094375

4. HELLER F, FLORIAN P, BOJARSKI C, et al. Interleukin-13 Is the Key Effector Th2 Cytokine in Ulcerative Colitis That Affects Epithelial Tight Junctions, Apoptosis, and Cell Restitution. *Gastroenterology*. 2005;129(2):550-564. doi:10.1016/j.gastro.2005.05.002

5. Mardini HE, Kip KE, Wilson JW. Crohn’s disease: a two-year prospective study of the association between psychological distress and disease activity. *Dig Dis Sci*. 2004;49(3):492-497. http://www.ncbi.nlm.nih.gov/pubmed/15139504. Accessed August 21, 2018.

6. Bitton A, Sewitch MJ, Peppercorn MA, et al. Psychosocial determinants of relapse in ulcerative colitis: a longitudinal study. *Am J Gastroenterol*. 2003;98(10):2203-2208. doi:10.1111/j.1572-0241.2003.07717.x

7. Winterkamp S, Weidenhiller M, Otte P, et al. Urinary excretion of N-methylhistamine as a marker of disease activity in inflammatory bowel disease. *Am J Gastroenterol*. 2002;97(12):3071-3077. doi:10.1111/j.1572-0241.2002.07028.x

8. Brzozowski B, Mazur-Bialy A, Pajdo R, et al. Mechanisms by which Stress Affects the Experimental and Clinical Inflammatory Bowel Disease (IBD): Role of Brain-Gut Axis. *Curr Neuropharmacol*. 2016;14(8):892-900. http://www.ncbi.nlm.nih.gov/pubmed/27040468. Accessed August 21, 2018.

9. Barclay GR, Turnberg LA. Effect of cold-induced pain on salt and water transport in the human jejunum. *Gastroenterology*. 1988;94(4):994-998. doi:10.5555/URI:PII:0016508588905586

10. Barclay GR, Turnberg LA. Effect of psychological stress on salt and water transport in the human jejunum. *Gastroenterology*. 1987;93(1):91-97. http://www.ncbi.nlm.nih.gov/pubmed/3582919. Accessed July 8, 2018.

11. Santos J, Saunders PR, Hanssen NP, et al. Corticotropin-releasing hormone mimics stress-induced colonic epithelial pathophysiology in the rat. *Am J Physiol*. 1999;277(2 Pt 1):G391-9. http://www.ncbi.nlm.nih.gov/pubmed/10444454. Accessed July 3, 2018.

12. Nink M, Krause U, Lehnert H, Beyer J. Safety and side effects of human and ovine corticotropin-releasing hormone administration in man. *Klin Wochenschr*. 1991;69(5):185-195. http://www.ncbi.nlm.nih.gov/pubmed/2033913. Accessed July 16, 2018.

13. J.Yeo K-T, Babic N, Hannoush ZC, Weiss RE. *Endocrine Testing Protocols: Hypothalamic Pituitary Adrenal Axis*. MDText.com, Inc.; 2000. http://www.ncbi.nlm.nih.gov/pubmed/25905177. Accessed July 16, 2018.

14. Pei R, DiMarco DM, Putt KK, et al. Premeal Low-Fat Yogurt Consumption Reduces Postprandial Inflammation and Markers of Endotoxin Exposure in Healthy Premenopausal Women in a Randomized Controlled Trial. *J Nutr*. 2018;148(6):910-916. doi:10.1093/jn/nxy046

15. Saunders PR, Kosecka U, McKay DM, Perdue MH. Acute stressors stimulate ion secretion and increase epithelial permeability in rat intestine. *Am J Physiol Liver Physiol*. 1994;267(5):G794-G799. doi:10.1152/ajpgi.1994.267.5.G794

16. Lee HS, Kim D-K, Kim YB, Lee KJ. Effect of acute stress on immune cell counts and the expression of tight junction proteins in the duodenal mucosa of rats. *Gut Liver*. 2013;7(2):190-196. doi:10.5009/gnl.2013.7.2.190

17. Costantini TW, Krzyzaniak M, Cheadle GA, et al. Targeting α-7 nicotinic acetylcholine receptor in the enteric nervous system: a cholinergic agonist prevents gut barrier failure after severe burn injury. *Am J Pathol*. 2012;181(2):478-486. doi:10.1016/j.ajpath.2012.04.005

18. Demaude J, Salvador-Cartier C, Fioramonti J, Ferrier L, Bueno L. Phenotypic changes in colonocytes following acute stress or activation of mast cells in mice: implications for delayed epithelial barrier dysfunction. *Gut*. 2006;55(5):655-661. doi:10.1136/gut.2005.078675

19. Vanuytsel T, van Wanrooy S, Vanheel H, et al. Psychological stress and corticotropin-releasing hormone increase intestinal permeability in humans by a mast cell-dependent mechanism. *Gut*. 2014;63(8):1293-1299. doi:10.1136/gutjnl-2013-305690

20. Uhde M, Ajamian M, Caio G, et al. Intestinal cell damage and systemic immune activation in individuals reporting sensitivity to wheat in the absence of coeliac disease. *Gut*. 2016;65(12):1930-1937. doi:10.1136/gutjnl-2016-311964

21. Pelsers MMAL, Namiot Z, Kisielewski W, et al. Intestinal-type and liver-type fatty acid-binding protein in the intestine. Tissue distribution and clinical utility. *Clin Biochem*. 2003;36(7):529-535. http://www.ncbi.nlm.nih.gov/pubmed/14563446. Accessed October 24, 2018.

22. Bode L, Salvestrini C, Park PW, et al. Heparan sulfate and syndecan-1 are essential in maintaining murine and human intestinal epithelial barrier function. *J Clin Invest*. 2008;118(1):229-238. doi:10.1172/JCI32335

23. Yablecovitch D, Oren A, Ben-Horin S, et al. Soluble Syndecan-1: A Novel Biomarker of Small Bowel Mucosal Damage in Children with Celiac Disease. *Dig Dis Sci*. 2017;62(3):755-760. doi:10.1007/s10620-016-4415-8

24. Yablecovitch D, Stein A, Shabat-Simon M, et al. Soluble Syndecan-1 Levels Are Elevated in Patients with Inflammatory Bowel Disease. *Dig Dis Sci*. 2015;60(8):2419-2426. doi:10.1007/s10620-015-3589-9

25. Schumann RR, Kirschning CJ, Unbehaun A, et al. The lipopolysaccharide-binding protein is a secretory class 1 acute-phase protein whose gene is transcriptionally activated by APRF/STAT/3 and other cytokine-inducible nuclear proteins. *Mol Cell Biol*. 1996;16(7):3490-3503. doi:10.1128/mcb.16.7.3490

26. Fraser JRE, Gibson PR. Mechanisms by which food intake elevates circulating levels of hyaluronan in humans. *J Intern Med*. 2005;258(5):460-466. doi:10.1111/j.1365-2796.2005.01564.x
