# Supplementary material for: Characterisation of biomarkers of intestinal barrier function in response to a high fat/high carbohydrate meal and corticotropin releasing hormone
Source: PLoS One. 2024 Feb 26;19(2):e0294918. doi: 10.1371/journal.pone.0294918 (PMC10896497; doi:10.1371/journal.pone.0294918)
Supplement: Attachment — Submitted filename: Reply to PLOS1 reviewer final.docx [file pone.0294918.s006.docx]

**Reply to reviewer**

We thank the reviewer for their comments and made have the following comments and changes to the manuscript.

This paper looks at a crossover trial of CRH vs placebo injections in people eating low fat low-carb meals.

The abstract is not entirely clear as the final HFHC meal is given first in a list of three interventions. The abstract needs to explain the study conduct in the same way as the methods as at present it is unclear from the abstract what is going on.

*REPLY: Thank you for this comment. This has now been amended in the abstract for clearer representation of the fact that the HFHC meal was given as the last intervention.*

The introduction and methods should explain the relevance of a crossover design here, and of the final shared HFHC meal, as this is not given clearly.

*REPLY: Thank you – this has been amended in the introduction section for clarity.*

Please explain why 10 volunteers was considered sufficient -what sort of differences were expected to be seen here?

*REPLY: Given the exploratory nature of this study and its intensive protocol, 10 participants who acted as their own controls was considered to be sufficient to elicit signals in intestinal permeability changes associated with present interventions.*

How was protocol compliance in terms of eating checked here?

*REPLY: Compliance with meal intake was checked in person by the study doctor by specific questioning of every participant at the time of each interaction. This point is now clarified in the study protocol section.*

The abstract should give actual p-values, and where possible effect sizes and confidence intervals.

*There were p values listed for each of the outcomes in the present manuscript. If further clarification is required please let us know.*

Why are only some timepoints tested in Figure 2? Please use a proper repeated measures model here - the statistical methods proposed are not appropriate - there are methods for doing this which are well known and documented. At present there is selective reporting and no adjustment for multiple testing.

*Thank you for this feedback.*

*REPLY: A clearer rationale for the timepoints chosen is now described on page 7 with addition of the following sentence:*

*These time points were chosen based on previous studies and were thought to be most representative of the changes seen in intestinal permeability biomarkers over a 24-hour period.*

*The statistical evaluation has been reworked with our statistician, Dr Frank Parker. Statistical comparisons were performed between relevant data. For instance, changes in biomarkers after CRH were not compared with those after the high-fat high-carbohydrate meal as this was not relevant to the questions being asked. The changes in the pre-intervention period were analysed separately.*

*The changes from baseline of the biomarkers were compared across the 2, 6 and 24-hour timepoints were compared across Friedman test or ANOVA where appropriate for repeated measures. Pairwise comparison between the control and CRH arms and between Control and HFHC arms were also performed since the interventions were acute (effects at 24 hours were not anticipated) and the individual timepoints are relevant to future studies where only one timepoint is usually performed. Hence, Figures 4 and 5 show these pairwise comparisons. Corrections were made for multiple comparisons where appropriate using the Bonferroni method.*

*We reassure the Reviewer that we are not selectively reporting the data. Scatterplots were shown to reveal all data where possible.*
