# Supplementary material for: Characterisation of biomarkers of intestinal barrier function in response to a high fat/high carbohydrate meal and corticotropin releasing hormone
Source: PLoS One. 2024 Feb 26;19(2):e0294918. doi: 10.1371/journal.pone.0294918 (PMC10896497; doi:10.1371/journal.pone.0294918)
Supplement: Attachment — Submitted filename: Reply to PLOS1 Oct 23.docx [file pone.0294918.s007.docx]

**Reply to reviewer**

We thank the reviewer for their comments and made have the following comments and changes to the manuscript.

Reviewer #1: This is a well-written report on a very small study of plasma concentrations of CORTICOTROPIN RELEASING HORMONE. This claims to be a "pilot" or "exploratory" clinical trial that shows that concentrations are not stable. The study does not consider power, and claim that "10 participants who acted as their own controls was considered to be sufficient to elicit signals in intestinal permeability changes associated with present interventions." I don't know what "elicit signals" means statistically, but it sounds like the authors are claiming adequate power to detect intestinal permeability changes. This needs to be clarified carefully in a statistical manner, because a low powered study, even if it achieves statistical significance in some metric, is not sufficient for statistical validity. Please clarify.

*Thank you for this feedback. The sample size was chosen based on previously published dietary studies in the intestinal permeability field which also used small numbers of participants. The study was designed to explore if a signal could be detected in the changes to intestinal permeability utilising the two approaches of intestinal barrier perturbation. This point has been clarified in the manuscript in the participants section but was also previously addressed in the manuscript – in the discussion section.*

Second, an "exploratory" or "pilot" study indicates that something will follow. What are the implications for future studies? That is the role of pilot studies, not to definitively show an outcome.

*Thank you for this feedback. The discussion section has been amended to add in this important point – “Despite its small sample size, this study provides guidance in designing larger studies utilise plasma markers of intestinal permeability.”*

The CONSORT document requires stating the randomization procedure used (blocks with block size, complete (simple randomization, etc.) Patients "were randomised ([www.randomisation.com](http://www.randomisation.com/)) to receive either a CRH or placebo injection" is not a randomization procedure.

*Thank you for this feedback. The paper has now been amended to state: “randomised to receive either a CRH or placebo injection first. This was calculated by using randomly computed blocks with random block sizes generated by the website www.randomisation.com.”*

Statistical analysis is adequate. No comments there.

Reviewer #2: Comments: This is an amazing work. But there are some area that should be re-visited.
1.The Sample size is Scanty, more information would have been obtained with a bigger sample size statistically.

*Thank you for this comment. We agree; however, the significant face-to-face involvement required by participants in the study in the setting of the pandemic precluded a larger number of participants. This limitation is discussed at length in the discussion.*

2. Gender population, 50% female I believe the biomarker would have had a different effects on different gender when compared, may be comparing the female and the male genders with regards to the biomarker.

*Thank you for this feedback. This was analysed for in the preliminary analysis with no differences between sexes detected. However, as above, we acknowledge the limitation of small participant numbers. Especially when looking at subgroup analyses.*

3.Sample Containers for specimen collection, EDTA was used for both analyte with protein back ground such as LBP and IFABP assay, Check the appropriate containers for the sample collections.

*Thank for this feedback. The samples were collected into contains which had previously been used for this analysis. A reference has been added to the methods section for clarity.*
.

Reviewer #3: REVIEWER’S REPORT TO AUTHORS FOR PONE-D-23-16375
Dear Authors,
The following changes are recommended to be changed after review of the Article titled: “Time-dependent characterization of biomarkers of intestinal barrier function in response to a high-fat, high-carbohydrate meal and corticotropin releasing hormone – a randomized, placebo-controlled cross-over study in healthy humans”
Title: I suggest that the title should be modified to read:
Characterization of biomarkers of intestinal barrier function in response to high-fat/high carbohydrate meal and corticotropin releasing hormone in healthy humans. (20 words title). The randomized placebo-controlled study can be indicated as the type of study in materials and methods.

*This has been amended as suggested, thank you.*

Abstract: Only A should be capitalized in abstract, use bold type, 18pt font as indicated in the manual style.

*This has been corrected, thank you*

Line 40: Add released before over (sentence incomplete)

*I am not clear which sentence the reviewer is referring to. The sentence on the line 2 above the sentence in the comment below appears complete.*

Line 42-44: Rephrase to- This study aimed to examine the stability and pharmacokinetics of changes in plasma concentrations of intestinal Fatty-acid -binding -protein (IFABP), Lipopolysaccharide-binging –protein (LBP), soluble CD14, and Syndecan-1 after acute stress and high fat-high-carbohydrate meal.

*This has been amended – thank you for the wording suggestion.*

Line 49: Mention sample size used for each group after the last word interventions

*The same sample size was used for each intervention – this paragraph has been amended to clarify this point as the original wording was indeed confusing.*

Line 61: Introduction, Adhere to body formatting guidelines

*Corrected, thank you*

Line 91: Add – were after syndecan-1

*We are sorry, but do not understand this requested change.*

Line 95-97: Add “but’ before the time line. Rephrase, therefore, can be rapidly released on intestinal injury. The timeline……

*Thank you for drawing our attention to this problematic sentence – this has now been rephrased.*

Line 99: Sentence in lines 99-105 too long, use; to separate aims

*Amended as requested*

Line 111: Male or female aged between ….remove and

*Amended as requested*

In participants, be specific; male and female? How many recruits from each groups/gender? State your sample size, location of study sites, ethical approval, state study design/type, sampling technique? How were they prepared? Fasting or non-fasting? Which meal did they take the previous day and at what Time?

*Thank you – the participant question was addressed in the protocol. Ethics approval, study location sites, sampling technique, study design type, fasting and oral intake status are all addressed in the study protocol section of the manuscript.*

Study Protocols: Rewrite line 119 (After the assessment by the study doctor to ensure eligibility, informed consent was signed by the participants. Use “Figure 1”

*Thank you. This has been corrected.*

Line 128-129: What volume of normal saline?) They then had further blood tests after 2 and 6 hours during which they remained near the [department.in](http://department.in/) line 129

*Thank you. This was 10ml and has been clarified in the manuscript.*

Line 129 Rephrase: Further blood tests was done after 2 and 6 hours as they remained nearby the sampling site.

*Thank you for the suggestion – this has been amended.*

Line 139: First visit
Study protocol: Specify contents explained to them as LFLC, HFHC etc. from the beginning of the study or was the above mentioned calories the same as used in the beginning of the study?), in study protocol

*Thank you. Line 142 of the revised manuscript states regarding the LFLC diet: These were the only foods that they were able to consume during the study*

Line 172-176: This should be moved to the beginning of study protocols for clarity and simply indicate statistical tools and types used. Example, Wilcoxon ranked sign test etc. as stated below. Timepoints should be spaced as time points

*Thank you for this feedback, however we feel that the clinical aspect of the trial rather should be specified prior to the laboratory specifications so have left the order as such in the manuscript. The statistical analysis is specified in line 204 of the new manuscript as suggested in this comment.*

Statistical analysis: Lines 180-182 (Time points) should be spaced out. Clarity about the statistical tools used needed.

*Thank you for this feedback. The statistical methods are described in detail in lines 212 to 225 of the revised manuscript.*

Line 196: Were females(Plural)

*Thank you for this feedback. We feel ‘one half were female’ rather than ‘one half were females’ may read nicer.*

Lines 204-208: Time points, see lines 204, 208 correct throughout

*Thank you. This has been amended in the manuscript as suggested.*

Line 211: “Figure 3” ; Always represent figure in parentheses, see others.

*Thank you. This has been amended in the manuscript.*

Line 252- this marked point should go to type of study in methodology. In this study.
Line 280; Un-fasted state
Line 282: Remove observed highlighted. Repeated words
Line 302: Use the sentence, this is in line with
Line 327: Rephrase to and a standard caloric intake must be ensured the day prior to measurement.

*Thank you for this feedback. Most of these points have been amended in the manuscript.*

References: Abide by the Journal manual reference style. Ensure in all, the recommended style is followed.

Additional Information: Authors should add the tables and figures as attachments. Authors are required to make all data underlying the findings described fully available, without restriction

*Thank you, this has been revised in this format.*

Authors should enter a financial disclosure statement that describes the sources of funding for the work included in this submission. Review the submission guidelines for detailed requirements. View published research articles from PLOS ONE for specific examples. This statement is required for submission and will appear in the published article if the submission is accepted. Please make sure it is accurate

Unfunded studies Enter: The author(s) received no specific funding for this work. Funded studies Enter a statement with the following details: Initials of the authors who received each award • • Grant numbers awarded to each author • The full name of each funder • URL of each funder website Did the sponsors or funders play any role in the study design, data collection and analysis, decision to publish, or preparation of the manuscript? • NO - Include this sentence at the end of your statement: The funders had no role in study design, data collection and analysis, decision to publish, or preparation.
NB: Please provide the Figures and tables for the work .
.

Reviewer #4: -The method and the results do not clearly address the first aim/ objective of the study,
-The diurnal variation of IFABP over the 24hrs which was one of the key findings was not properly described
-The study had low sample size and the age difference of the participants were wide apart. this may have affected the results

*Thank you for this feedback – the issue of the small sample size has been addressed in the manuscript.*

-The cross study design may not be appropriate in this type of study, as the effect of first intervention may affect the second intervention

*Thank you for this feedback. We feel the washout period of 2 weeks a significant duration of time to wash out any effect of corticotropin releasing hormone. The half-life of this is less than 10 minutes and the intestinal barrier can replenish itself in under 1 week. Analysis of the responses to CRH were similar whether it or saline were used first. This is now included in the results.*

The author should state clearly how long it took for the study, this will help to determine the level of compliance of the participants and validity of results

*Thank you for this feedback. The times taken for each arm and visit of the study are presented in “figure 1” - flowchart of the study.*

-The results were presented at different P values which may have affected the interpretation of the results even though they were corrected

*Thank you for this feedback – the correction was felt necessary by our statistician and previous reviewers.*

-The figures were not self explanatory, another format of line diagram that clearly shows the levels of the biomarkers across the 24hrs may be better

*Thank you for this feedback. The legends have been expanded. The selected time points were chosen to draw attention to the specific time points. We had previously expressed the markers across a 24-hour time frame but the graphs lacked clarity when presented in such a manner.*

- The author should clearly state the basis for the time point interval

*Thank you – this has been amended in the revised manuscript.*

-The author should objectively discuss the results obtained from the study
-The use of ambiguous statement in result presentation like" There was a tendency for reduction inn plasma LBP at 2 hours after CRH injection compared with that after the saline injection ......" should be revised

*The results are now objectively described.*

-some other grammatical errors should also be corrected
